# Determinants of neonatal mortality at neonatal intensive care unit in Northeast Ethiopia: unmatched case-control study

**DOI:** 10.1186/s41182-020-00232-9

**Published:** 2020-06-03

**Authors:** Abebaw Yeshambel Alemu, Getaneh Mulualem Belay, Mengistu Berhanu, Biniam Minuye

**Affiliations:** 1 Department of Nursing, College of Health Science, Debre Tabor University, Debre Tabor, Ethiopia; 2 Department of Pediatric and Child Health Nursing, School of Nursing, College of Medicine and Health Sciences, University of Gondar, P.O.Box:196, Gondar, Ethiopia

**Keywords:** Neonatal mortality, Neonatal factors, Neonatal intensive care unit, Northeast Ethiopia

## Abstract

**Background:**

Globally, in 2016, about 38% and 3% of all neonatal death were recorded in sub-Saharan Africa and Ethiopia, respectively. In the same year, 47 neonates out of 1000 live births were not surviving in the first 28 days of age in the Amhara region, Ethiopia. Despite the highest burden of neonatal death in the region, specific or the proximate determinants of neonatal death in the neonatal intensive care unit were not well identified.

**Objective:**

This study aimed to identify the determinants of neonatal mortality at neonatal intensive care unit in Dessie Referral Hospital, Northeast Ethiopia.

**Methods:**

An institution-based unmatched case-control study was conducted on neonates admitted to the neonatal intensive care unit of Dessie Referral Hospital, from January 1, 2016, to December 30, 2017. A total of 390 charts (130 cases and 260 controls) were selected by simple random sampling technique. The data were abstracted from the facility-based data abstraction form. A binary logistic regression analysis was fitted to identify the determinants of neonatal mortality.

**Results:**

Pregnancy-induced hypertension (AOR = 4.57; 95% CI 1.45–14.43), prolonged rupture of membrane (AOR = 2.04; 95% CI 1.13–3.68), very low birth weight (AOR = 7.00; 95% CI 2.10–23.35), and low birth weight (AOR = 2.12; 95% CI 1.10–4.20) were identified factors. Moreover, respiratory distress syndrome (AOR = 3.61; 95% CI 1.10–12.04), perinatal asphyxia (AOR = 2.27; 95% CI 1.18–4.39), meconium aspiration syndrome (AOR = 2.35; 95% CI 1.12–4.97), and infection (AOR = 2.26; 95% CI 1.34–3.82) were also significantly associated with neonatal death.

**Conclusions:**

Pregnancy-induced hypertension, prolonged rupture of membrane, low birth weight, respiratory distress syndrome, perinatal asphyxia, meconium aspiration syndrome, and infections were the major determinants of neonatal mortality. Therefore, special attention will be given to small and sick babies. Moreover, early anticipation of complications and management of mothers who had pregnancy-induced hypertension and prolonged rupture of the membrane would save neonates.

## Background

Neonatal mortality is defined as neonate who was born alive after 28 weeks of gestational age and died within the first 28 days [1]. In 2016, about 7000 and 2.6 million neonates died daily and per annum in the world, respectively. Of those, Southern Asia and sub-Saharan Africa dually hosted nearly 80%. Specifically, 38% and 3% were in sub-Saharan Africa and Ethiopia, respectively [2]. Among 194 countries worldwide including Ethiopia, preterm birth complications, intrapartum complications, and infections were the major determinants of neonatal death [3]. Previously, integration of Reproductive Maternal Newborn and Child Health (RMNCH), policy formulation, strong leadership, and partnership, as well as evidence-based interventions have reduced neonatal mortality from 49 to 40% worldwide in 2016 [2]. Besides, using a standard neonatal care protocol since 2014 [4] and free maternal and neonatal health services had a paramount advantage in Ethiopia [5]. According to the Ethiopian Demographic and Health Survey (EDHS, 2016) report, neonatal mortality in Ethiopia had been reduced by 41% due to the standardized neonatal care and free MCH services since 2014 [6].

Maternal, neonatal, intrapartum, and contextual factors were the major determinants of neonatal mortality worldwide [7]. Among maternal determinants that positively associated with early neonatal death were antepartum hemorrhage, pregnancy-induced hypertension, and other medical/surgical conditions [8]. Primiparity has increased the risk of neonatal deaths in Cameroon neonatal intensive care unit (NICU) [9]. Additionally, maternal age less than 20 years was positively associated with neonatal death in Brazil [10]. Besides, no antenatal care (ANC) visit was identified as a factor of early neonatal mortality in Ethiopia [11].

Moreover, a study conducted in the NICU of Cameroon revealed intrapartum factors like prolonged rupture of membrane (> 12 h) and home delivery were positively associated with neonatal death while institutional and cesarean delivery was negatively associated with neonatal death [9]. Another study conducted in the NICU of Kenya showed that mal-presentation (dystocia) was increasing the risk of early neonatal death [12]. Likewise, home delivery was larger for cases than controls in a study conducted in Indonesia [13]. Furthermore, a study conducted in Ethiopia revealed instrument delivery had increased the risks of neonatal death [14].

Neonatal death was significantly associated with low birth weight (LBW) [9, 10], very low birth weight (VLBW) [15, 16], extreme low birth weight (ELBW) [15], gestational age less than 37 weeks [10, 16], neonatal sepsis [9, 15], perinatal asphyxia [9], and congenital malformations [9, 10, 16]. Besides, one of the Indonesia studies added male sex as a positively associated risk factor [13].

One study conducted in Ethiopia teaching referral hospitals indicated that early neonatal death was positively associated with low birth weight babies who were born preterm [11]. Besides, neonatal sepsis [14, 17], perinatal asphyxia, respiratory distress syndrome, severe hypothermia [14], respiratory distress syndrome, and LBW [17] increased the risk of neonatal mortality. Additionally, multiple pregnancies were also a risk for early neonatal mortality [11].

Despite success in the past, Ethiopia is among the five highest neonatal death burden countries of the world in 2016 [2]. Locally, the Amhara region has the highest neonatal mortality (47/1000 live-birth) in the country [6]. In addition to this burden in the region, the prevalence of death in the NICU was 23.1% [17].

To overcome the burden of neonatal mortality, different strategies were implemented, including launching the Health Sector Transformation Plan (HSTP) [18], expanding NICU services, and opening the neonatal nursing program. However, neonatal mortality in the country was not significantly reduced. As far as our searching, there are no similar studies that had been conducted in Ethiopia. Therefore, we conducted a case-control study to identify determinants (or most important cause) of neonatal mortality in the Amhara region to plan specific interventions to avert neonatal death.

## Methods and materials

### Study setting and period

The study was conducted at NICU in Dessie Referral Hospital from March 15 to April 15, 2018, which is located 401 km away from Addis Ababa, Northeast Ethiopia. The hospital is one of the referral-level hospitals in the region serving more than 8 million people, and it is the only referral hospital giving referral service for North Wollo zone, South Wollo zone, Oromia Special administrative zone, North Shewa Zone, and part of Afar and southern Tigray region. It was giving NICU service since September 2012. Currently, the unit has three sections, i.e., Kangaroo Mother Care (KMC), term, and preterm sections. The unit is equipped with 3 phototherapy machines, 4 incubators, 3 radiant warmers, 3 heaters, 2 oxygen concentrators, and 10 filled oxygen cylinders per 15 days. Besides, bubble continuous positive airway pressure (CPAP) prepared using locally available materials like ringer lactate bag, tap water, and oxygen cylinder was used. However, there is no automated CPAPA and mechanical ventilator.

According to the Ethiopian central statistics agency, the total population of the South Wollo zone is projected to be 3,034,327 (1,558,363 women) in 2016 [19]. Using 31.8/1000 population in Ethiopia [6], the crude live birth of the zone could be estimated to 96,492 births in 2016. Additionally, using 14.7% of all household populations are under-five children in Ethiopia in 2016 [6].

### Study design

An institution-based unmatched case-control study was conducted to identify determinants of neonatal mortality among NICU admissions of Dessie Referral Hospital.

### Selection of cases and controls

All NICU admissions in Dessie Referral Hospital from January 1, 2016, to December 30, 2017, who had a death record at discharge were included as cases. Besides, those who were discharged alive were included as controls.

### Exclusion criteria

Exclusion criteria for cases were unrecorded outcome at discharge, whereas for controls, all alive discharges with an unrecorded outcome and referrals and those who against medical advice were excluded. Moreover, records with incomplete data on gestational age and weight were excluded from either cases or controls.

### Sampling method

**Fig. 1:**
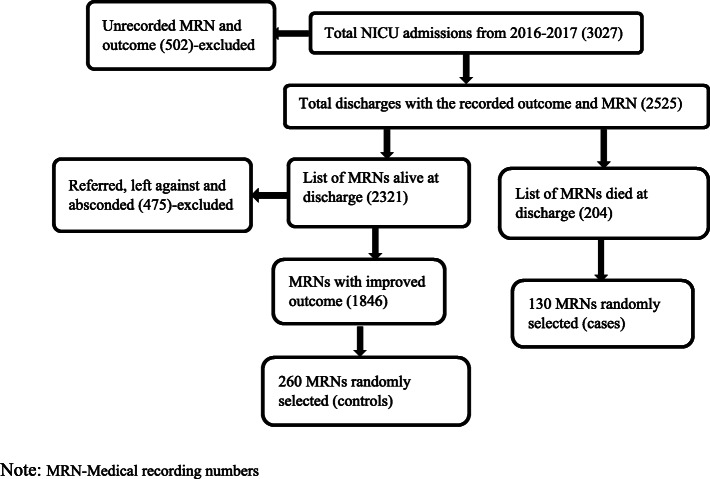
Sampling procedure to study determinants of death at Dessie Referral Hospital NICU from 2016 to 2017, in 2018. Note: MRN—medical recording numbers

The samples were taken among the admissions from January 1, 2016, to December 30,2017. The minimum sample size was determined using Epi Info 2013 version 7.1.2.0 after input of case to control ratio 1:2, power 80%, and confidence interval 95%. About 26.9% [17], 34.4%, and 51.2% [14] of controls were expected to be exposed for neonatal sepsis, perinatal asphyxia, and respiratory distress syndrome, respectively. The first minimum samples calculated using the software were 354, 329, and 339 orderly. The maximum size of 354 (118 cases and 236 controls) was taken. Then, 10% nonresponse rate was added for reviewed charts discarded because of an unrecorded outcome, gestational age, and weight [20]. Finally, the maximum size of 390 (130 cases and 260 controls) was taken. Using the inclusion criteria, the sampling frame was prepared for cases and controls from a serial list of neonates’ medical record numbers, which were taken from integrated admission and discharge logbooks in the NICU. Then, using a simple random sampling technique, 390 neonates’ medical record numbers were taken to extract their charts from the hospital archive room. The medical recording numbers of sample charts were selected using the procedure in Fig. 1.

### Operational definition

Neonatal infection—any recorded neonatal condition in the chart from any of following: tetanus, sepsis, pneumonia, meningitis, syphilis, diarrhea, and others that has been confirmed by a bacteriological laboratory [21].

Maternal and labor and delivery determinants**—**any recorded maternal condition was extracted from the chart in NICU [12].

Neonates’ weight**—**grouped into ELBW (< 1000 g), VLBW (1000–1499 g), LBW (1500–2499 g), normal birth weight (NBW) (2500–3999 gram), and macrosomia (≥ 4000 g) [4].

Gestational age—age of the neonate lapsed in inception determined from LNMP, Ballard, or ultrasound [1], then grouped into preterm (< 37 weeks), term (37–42 weeks), and > 42 weeks [4].

Prolonged rupture of membrane—in this study, prolonged rupture of membrane is defined as a rupture of membrane lasting longer than 12 h before labor begins [9]**.**

### Data collection

Ethiopian Public Health Institute (EPHI) facility-based perinatal death surveillance data abstraction format with 8 parts [22] was adapted and modified. Then, the abstraction form was designed in 4 parts: general information of the neonate and the mother, antenatal history of the mother, labor and delivery history, and postnatal history of the neonate.

Two BSc nurses, who were working at NICU in Dessie Referral Hospital, abstracted secondary data from 390 neonate charts. The data on maternal, neonatal, labor, and delivery factors were abstracted from medical records.

Data abstraction form was tested on 10% of sample neonate charts (13 cases and 26 controls) at NICU in Woldia General Hospital found in North Wollo zone. Then, data abstraction form was modified into maternal, labor and delivery, and neonatal factors. Since contextual and delay factors were not recorded during the pretest, they were removed from the data collection tool.

Each chart was coded to avoid errors during data collection. Besides, 2 BSc nurse data collectors and 1 general practitioner supervisor were trained for 1 day on data collection and abstraction from the charts. Since no data was interviewed from the mother, the English version of the data abstraction form was used. Moreover, the completed data format was checked daily for completeness. Finally, the data were entered into Epi info version 4.2 and exported to Stata version 14 for further analysis.

### Data analysis

Continuous data were summarized by mean and SD, and a *t* test was conducted to compare between two groups. Additionally, complete case analysis was done because all variables used in regression analysis had missing data less than 10%; the frequency of missing data was computed. All categorical variables with less than five observations were merged in simple and multiple binary logistic regression analysis.

Since most data on tetanus toxoid (TT) vaccination, iron supplementation, and level of the facility for ANC visits were not recorded in charts, they were removed from further analysis. On the other hand, age in days during admission and discharge, level of the facility for outborn, other neonatal conditions, and duration of labor were presented for descriptive purposes only.

To admit more determinants in the multivariable logistic regression model, statistically significant variables at *p* value ≤ 0.2 in bivariable logistic regression were used. The crude odds ratio (COR) with a 95% confidence level was reported for the bivariable logistic regression model. Since the Hosmer-Lemeshow *p* value was 0.502, the final multivariable logistic regression model was well fitted at *p* value > 0.05. Finally, an adjusted odds ratio (AOR) with a 95% confidence level at *p* value < 0.05 was used to declare an association between the outcome variable and determinant variables.

## Results

A total of 390 (130 cases and 260 control) neonate charts were reviewed during the study period. Out of all charts reviewed, about 6 (4 cases and 2 control) abstracted data forms were discarded because their weight was unrecorded in three (2 cases and 1 control) and gestational age in the remaining. The proportion of missing data was ranged from 0.8 to 11.9% across variables.

### Maternal characteristics

In this study, the majority (86.4%) of cases (deaths) were delivered from mothers between 20–34 years of age. More than half (52.4%) of deaths were the first liable babies (parity 1). About 109 (86.5%) of mothers among the case group had at least ANC visits. Most deaths were delivered from mothers who had no hypertensive disorder (88.9%) and medical complications (90.0%). Additionally, in both series, more than three fourth of babies were delivered in the health facility. However, nearly two-thirds were inborn in both groups. About one fourth of babies studied were delivered by cesarean section in this study. Unlike controls (17%), more cases (29%) were delivered later than 12 h rupture of membrane. Moreover, around 64.8%, 10.2%, and 25% of cases were delivered by spontaneous vaginally (SVG), assisted, and cesarean section, respectively. However, in controls, SVG, assisted, and cesarean section were 59.5%, 15.2%, and 26.3%, respectively (Table 1).

**Table 1:** Maternal characteristics of the neonates admitted to neonatal intensive care unit of Dessie Referral Hospital, Northeast Ethiopia, 2017/2018 (*n* = 384)

Variables	Cases (***n*** = 126) (%)	Controls (***n*** = 258) (%)	Total (%)
**Maternal age**			
< 20	9 (7.1)	13 (5.0)	22 (5.8)
20–34	108 (86.4)	229 (88.8)	337 (88.2)
34–45	8 (6.4)	16 (6.0)	23 (6.0)
**Parity**			
Primi-para (parity 1)	66 (52.4)	151 (58.5)	217 (56.7)
Multi-para ( parity 2–4)	52 (41.3)	94 (36.4)	146 (38.1)
Grand multiparty (parity ≥ 5)	7 (5.6)	13 (5.0)	20 (5.2)
**ANC visit**			
Yes	109 (86.5)	220 (85.3)	329 (86.4)
No	16 (12.7)	30 (12.8)	46 (12.1)
Unrecorded	1 (0.8)	5 (1.9)	6 (1.6)
**Hypertensive disorders of pregnancy**			
Yes	14 (11.1)	6 (2.3)	20 (5.2)
No	112 (88.9)	252 (97.3)	364 (94.8)
**Maternal hemorrhage (antepartum hemorrhage)**			
Yes	12 (9.5)	7 (2.7)	19 (4.9)
No	114 (90.5)	251 (97.3)	365 (95.1)
**Other medical/surgical conditions**			
Yes	5 (4.0)	15 (5.8)	20 (5.2)
No	121 (90.0)	243 (94.2)	364 (94.8)
**Place of delivery**			
Home	18 (14.3)	34 (13.2)	52 (13.6)
Facility	108 (85.7)	224 (86.8)	332 (86.5)
**Place of birth for facility deliveries**			
Inborn	64 (59.3)	145 (64.7)	209 (63.0)
Outborn	44 (40.7)	79 (35.3)	123 (37.1)
**Level of the facility for outborn**			
Health center	48 (80.0)	65 (68.4)	113 (72.9)
Hospital	6 (10.0)	15 (15.8)	21 (13.6)
Private health facility	6 (10.0)	15 (15.8)	21 (13.6)
**Mal-presentation**			
Yes	14 (11.1)	21 (8.1)	35 (9.1)
No	112 (88.9)	237 (91.9)	349 (90.9)
**Duration of rupture of membrane**			
≤ 12 h	82 (65.1)	189 (73.3)	271 (77.4)
> 12 h	36 (28.6)	43 (16.7)	79 (22.6)

Some variables may not sum up 384 because those variables might not be recorded but not discarded from further analysis except gestational age and weight. Other medical/surgical conditions (chronic hypertension, diabetes mellitus, HIV/AIDS, infections, etc.)

### Neonatal characteristics

Of all 384 neonates (126 cases and 258 controls) understudy, more than half 86 (68.3%) cases and 165 (64.0%) controls were male. Besides, more than three fourth 113 (89.7%) cases and 226 (87.6%) controls were singleton babies. Similarly, the majority 112 (88.9%) cases and most 239 (92.6%) controls were admitted before 7 days of life. Moreover, about 89 (70.6%) deaths among cases occurred in early life and 66.3% of improved discharges were before 7 days of age (Table 2).

**Table 2:** Neonatal characteristics that admitted to neonatal intensive care unit of Dessie Referral Hospital, Northeast Ethiopia, 2017/2018 (*n* = 384)

Variables	Cases (***n*** = 126) (%)	Controls (***n*** = 258) (%)	Total ***n*** (%)
**Neonates’ sex**			
Male	86 (68.3)	165 (64.0)	251 (65.4)
Female	40 (31.8)	93 (36.1)	133 (34.6)
**Age at admission**			
≤ 7 days	112 (88.9)	239 (92.6)	351 (91.4)
> 7 days	14 (11.1)	19 (7.4)	33 (8.6)
**Age at discharge**			
≤ 7 days	89 (70.6)	117 (66.3)	260 (67.7)
> 7 days	37 (29.4)	87 (33.7)	124 (32.3)
**Type of birth**			
Singleton	113 (89.7)	226 (87.6)	339 (88.3)
Multiple	13 (10.3)	32 (12.4)	45 (11.7)
**Gestational age in completed weeks**			
< 37	44 (34.9)	63 (24.4)	107 (27.9)
≥ 37	82 (65.1)	195 (75.6)	277 (72.1)
**Weight at admission in grams**			
700–1499	24 (19.0)	14 (5.4)	38 (9.9)
1500–2499	43 (34.1)	72 (27.9)	115 (30.0)
≥ 2500	59 (46.8)	172 (66.7)	231 (60.2)
**Perinatal asphyxia**			
Yes	40 (31.8)	37 (14.3)	77 (20.1)
No	86 (68.3)	221 (85.7)	307 (79.9)
**Meconium aspiration syndrome**			
Yes	24 (19.1)	23 (8.9)	47 (12.2)
No	102 (81.0)	235 (91.1)	337 (87.8)
**Congenital anomalies**			
Yes	12 (9.5)	14 (5.4)	26 (6.8)
No	114 (90.5)	244 (94.6)	358 (93.2)
**Respiratory distress syndrome**			
Yes	16 (12.7)	8 (3.1)	24 (6.3)
No	110 (87.3)	250(96.9)	360 (93.8)
**Infection (sepsis, meningitis, pneumonia)**			
Yes	78 (61.9)	109 (42.3)	187 (48.7)
No	48 (38.1)	149 (57.8)	197 (51.3)
**Hypothermia at admission**			
Yes	65 (51.6)	116 (45.0)	181 (51.1)
No	61 (48.4)	112 (55.0)	173 (48.9)
**Hypoglycemia**			
Yes	10 (7.9)	7 (2.7)	17 (4.4)
No	116 (92.1)	251 (97.3)	367 (95.6)
**Other neonatal conditions**			
Yes	24 (19.1)	39 (15.1)	63 (16.4)
No	102 (81.0)	219 (84.9)	321 (83.6)

Some variables may not sum up 384; other neonatal conditions (necrotizing enterocolitis, anemia, HIV exposed, hemolytic disease of the newborn, birth trauma, jaundice, polycythemia, hepatitis exposed, omphalitis)

In this study, the mean gestational age of the case was 36^+1^ weeks, for controls 37^+3^ weeks. Cases had a mean weight of 2284 g, while their counterparts had 2644 g. Additionally, mean maternal age and parity were 25 years and 2 births, respectively, which was the same for cases and controls. Nonetheless, the mean duration of the rupture of the membrane among cases was 9.6 h, about 2.4 h lager from the control series (*p* < 0.01) (Table 3).

**Table 3:** Summary statistics of the neonates and maternal characteristics that admitted to neonatal intensive care unit of Dessie Referral Hospital, Northeast Ethiopia, 2017/2018 (*n* = 384)

Variable	Cases (***n*** = 126)		Controls (***n*** = 258)		***p*** value
	Mean	SD	Mean	SD	
Maternal age (years)	26	4	26	4	0.4
Maternal parity	2	1	2	1	0.8
DROM (days)	10	14	7	13	< 0.01
DOL (hours)	13	11	10	6	1
Gestational age (weeks)	36	3	37	2	< 0.01
Weight at admission (grams)	2284	816	2644	731	< 0.01

*DROM* duration of rupture of membrane, *DOL* duration of labor

### Determinants of neonatal death

The odds of death were more likely observed among neonates delivered from mothers who had pregnancy-induced hypertension (AOR = 4.57; 95% CI 1.45–14.43) and duration of rupture of membrane > 12 h before birth (AOR = 2.04; 95% CI 1.13–3.68). Meanwhile, compared with controls, cases had higher odds of perinatal asphyxia (AOR = 2.27; 95% CI 1.18–4.39), meconium aspiration syndrome (AOR = 2.35; 95% CI 1.12–4.97), respiratory distress syndrome (AOR = 3.61; 95% CI 1.10–12.04), VLBW (AOR = 7.00; 95% CI 2.10–23.35), LBW (AOR = 2.12; 95% CI 1.10–4.20), and infections (AOR = 2.26; 95% CI 1.34–3.82) (Table 4).

**Table 4:** Determinants of neonatal mortality that admitted to neonatal intensive care unit of Dessie Referral Hospital, Northeast Ethiopia, 2017/2018 (*n* = 384)

Variables	Cases (***n*** = 126) (%)	Controls (***n*** = 258) (%)	COR (95% CI)	AOR (95% CI)
**Hypertensive disorders of pregnancy**				
Yes	14 (11.1)	6 (2.3)	5.22 (1.95–13.96)	4.57 (1.45–14.43)**
No	112 (88.9)	252 (97.3)	1	1
**Maternal hemorrhage (antepartum hemorrhage)**				
Yes	12 (9.5)	7 (2.7)	3.75 (1.44–9.79)	3.28 (0.86–12.46)
No	114 (90.5)	251 (97.3)	1	1
**Duration of rupture of membrane**				
≥ 12 h	36 (28.6)	43 (16.7)	1.92 (1.15–3.22)	2.04 (1.13–3.68)*
< 12 h	82 (65.1)	189 (73.3)	1	1
**Gestational age in completed weeks**				
< 37	44 (34.9)	63 (24.4)	1.66 (1.04–2.64)	0.43 (0.17–1.10)
≥ 37	82 (65.1)	195 (75.6)	1	1
**Weight at admission in grams**				
700–1499	24 (19.0)	14 (5.4)	4.99 (2.42-10.29)	7.00 (2.10-23.35)^**^
1500–2499	43 (34.1)	72 (27.9)	1.74 (1.07-2.81)	2.12 (1.10-4.20)^*^
≥ 2500	59 (46.8)	172 (66.7)	1	1
**Perinatal asphyxia**				
Yes	40 (31.8)	37 (14.3)	2.77 (1.66–4.63)	2.27 (1.18–4.39)*
No	86 (68.3)	221 (85.7)	1	1
**Meconium aspiration syndrome**				
Yes	24 (19.1)	23 (8.9)	2.40 (1.29–4.54)	2.35 (1.12–4.97)*
No	102 (81.0)	235 (91.1)		1
**Congenital anomalies**				
Yes	12 (9.5)	14 (5.4)	1.83 (0.82–4.09)	1.89 (0.67–5.34)
No	114 (90.5)	244 (94.6)	1	1
**Respiratory distress syndrome**				
Yes	16 (12.7)	8 (3.1)	4.54 (1.88–10.93)	3.61 (1.10–12.04)*
No	110 (87.3)	250 (96.9)	1	1
**Infection (sepsis, meningitis, pneumonia)**				
Yes	78 (61.9)	109 (42.3)	2.22 (1.43–3.43)	2.25 (1.34–3.78)**
No	48 (38.1)	149 (57.8)	1	1
**Hypoglycemia**				
Yes	10 (7.9)	7 (2.7)	3.09 (1.14–8.32)	2.61 (0.77–8.79)
No	116 (92.1)	251 (97.3)	1	1

*Significant at *p* < 0.05

**Significant at *p* < 0.01

## Discussion

Despite the highest burden of neonatal death in the region, specific determinants of death in the neonatal intensive care unit (NICU) were not studied enough. Therefore, this study aimed to determine the determinants of neonatal mortality at neonatal intensive care unit in Dessie Referral Hospital. Consequently, pregnancy-induced hypertension, prolonged rupture of membrane, low birth weight, respiratory distress syndrome, perinatal asphyxia, meconium aspiration syndrome, and infection were significantly associated with neonatal death.

The odds of neonatal death were five times more likely among babies delivered from mothers who had pregnancy-induced hypertension as compared to those from no pregnancy-induced hypertension. Similar findings were reported previously from Ethiopia and the Netherlands [23, 24]. Since maternal hypertensive disorders of the pregnancy increased the risk of low birth weight, low 1st minute Apgar score, respiratory distress syndrome, and preterm birth, increased odds of death might be due to these effects of hypertensive disorders of pregnancy. Similarly, evidence showed preeclampsia increases the risk of small for gestational age delivery [24]; hence, babies born small for their gestational age are at an increased risk of neonatal death [25].

Among labor and delivery determinants, the odds of neonatal death from prolonged rupture of the membrane was two times higher for cases than controls. This finding is supported by a case-control study conducted in Cameroon [9]. Prolonged rupture of the membrane increases the risk ascending transmission of group B Streptococcus [26] and infection (sepsis) too [27]. This is recitation as sepsis increases the risk of neonatal death [9].

This study was also found the odds of neonatal death were seven times more likely for VLBW neonates. The finding is in line with previous studies done in Mexico [16], Uganda [15], and Ethiopia [11, 28]. Besides, despite a decrease in the strength of odds compared with VLBW, the odds of LBW was higher for cases than controls in our study. It is in agreement with studies done in Ethiopia [11, 28]. VLBW babies are more likely to be preterm, hypothermic, and develop necrotizing enterocolitis [29]. Neurologic pathologies like intraventricular hemorrhage and periventricular leukomalacia are common among babies who have below normal weight. Intraventricular hemorrhage might be complicated to intraventricular hemorrhagic infarction, while periventricular leukomalacia might end up with necrosis of the brain white matter [30]. All of these might lead to neonatal death.

Neonates who had respiratory distress syndrome were found three times more likely to die compared to their opposite wings. The same result was mentioned in Ethiopia [17]. Hyaline membrane formation incurs the lung less compliant to respiratory distress, so extra normal pressure is needed to inflate alveoli and small airways [30]. Hence, neonates will die due to the alveolar collapse of the noncompliant lung.

The odds of neonatal death from perinatal asphyxia was two times higher among cases than controls. This result is in line with studies done in Cameroon [9] and Ethiopia [11, 14, 31]. It might be due to a bad prognosis of asphyxia to hypoxic-ischemic encephalopathy [32] and renal system dysfunction [33]. Therefore, death could be portrayed as those bad prognostic results.

The odds of neonatal death were two times more likely observed among neonates who had an infection (sepsis, pneumonia, meningitis) aside from no infection. This finding is in agreement with the previous studies conducted in Ethiopia and Uganda [14, 15, 17], as infection (sepsis) is complicated to septic shock and multiple organ dysfunction, in which both are the most common causes of death in the neonatal period [34].

Meconium aspiration syndrome was determining the odds of death nearly three times. The result is supported by a study in India [35]. Since meconium aspiration syndrome entails chemical pneumonitis with pulmonary hypertension [36], and myocardial dysfunction [35], all these conditions might end up with death.

Therefore, the findings of this study will be an input to researchers as baseline to do further on neonatal mortality. Moreover, the findings of this study will be utilized by program implementers, decision, and policymakers to reduce neonatal mortality in the country. In general, investigating the determinants of neonatal death is meant to avert neonatal death.

## Limitation

Even though this study investigates the most important determinants of neonatal mortality, our study encountered limitations like missing information both on mother and neonates. For instance, economic status and contextual were not assessed. Moreover, medical record incompleteness is another main concern.

## Conclusion

Determinants of neonatal mortality in NICU of Dessie Referral Hospital, Northeast Ethiopia, were pregnancy-induced hypertension, prolonged rupture of membrane, VLBW, LBW, respiratory distress syndrome, perinatal asphyxia, meconium aspiration syndrome, and infection.

Based on findings, it would be important to give due attention for neonates diagnosed for perinatal asphyxia, meconium aspiration syndrome, respiratory distress syndrome, infections, and low birth weight. Besides, it would be better to strengthen the administration of intrapartum prophylactic antibiotics for laboring mothers with prolonged rupture of membrane and strengthen immediate newborn care for neonates delivered from mothers who had hypertensive disorders of pregnancy. Moreover, preconception care should be provided for mothers who had chronic hypertension.

## Data Availability

No additional data are required; all information is clearly stated in the main manuscript.

## Abbreviations

ANC: Antenatal care
AOR: Adjusted odds ratio
CI: Confidence interval
COR: Crude odds ratio
EDHS: Ethiopian Demographic and Health Survey
ELBW: Extreme low birth weight
HSTP: Health Sector Transformation Plan
KMC: Kangaroo mother care
LBW: Low birth weight
LNMP: Last normal menstrual period
MDG: Millennium Development Goal
NICU: Neonatal intensive care unit
VLBW: Very low birth weight
